# Serum afamin and its implications in adult growth hormone deficiency: a prospective GH-withdrawal study

**DOI:** 10.3389/fendo.2024.1348046

**Published:** 2024-02-06

**Authors:** Balázs Ratku, Hajnalka Lőrincz, Sára Csiha, Veronika Sebestyén, Eszter Berta, Miklós Bodor, Endre V. Nagy, Zoltán Szabó, Mariann Harangi, Sándor Somodi

**Affiliations:** ^1^ Institute of Health Studies, Faculty of Health Sciences, University of Debrecen, Debrecen, Hungary; ^2^ Department of Emergency Medicine, Faculty of Medicine, University of Debrecen, Debrecen, Hungary; ^3^ Doctoral School of Health Sciences, University of Debrecen, Debrecen, Hungary; ^4^ Division of Metabolism, Department of Internal Medicine, Faculty of Medicine, University of Debrecen, Debrecen, Hungary; ^5^ Department of Clinical Basics, Faculty of Pharmacy, University of Debrecen, Debrecen, Hungary; ^6^ Division of Endocrinology, Department of Internal Medicine, Faculty of Medicine, University of Debrecen, Debrecen, Hungary

**Keywords:** adult growth hormone deficiency, growth hormone withdrawal, afamin, body composition, insulin sensitivity, biomarker

## Abstract

**Introduction:**

Adult growth hormone deficiency (AGHD) is associated with a high prevalence of metabolic syndrome (MS), which contributes to the unfavorable cardiovascular risk profile in these patients. Insulin like growth factor-1 (IGF-1) is a widely used biomarker, however it does not always reflect the cardiometabolic risk and has a poor relationship with clinical efficacy endpoints. Consequently, there is an unmet need for biomarkers to monitor responses to GH-replacement. Afamin is a hormone-like glycoprotein, expressed in the liver. Higher afamin levels are strongly associated with MS and insulin resistance (IR). Although both MS and IR are very common in AGHD, afamin has not been investigated in these patients.

**Purpose:**

To investigate afamin as a potential biomarker in patients with AGHD.

**Materials and methods:**

Participants included 20 AGHD patients (11 GH-substituted and 9 GH-unsubstituted) and 37 healthy controls. Subjects underwent routine laboratory examinations, anthropometric measurements, body composition analysis using multi-frequency bioelectrical impedance analysis (InBody720) and measurement of serum afamin concentrations. In GH-substituted subjects, GH-substitution was withdrawn for 2 months. Measurements were carried out right before GH-withdrawal, at the end of the 2-month withdrawal period, and 1 month after reinstituting GH-replacement therapy (GHRT).

**Results:**

GH-unsubstituted patients demonstrated higher afamin levels compared to controls (p=0.03). Afamin positively correlated with skeletal muscle mass, bone mineral content, total body water, extracellular- and intracellular water content, insulin (all, p<0.01), HOMA-IR (p=0.01) and C-peptide (p=0.03) levels in AGHD but not in healthy controls. In GH-substituted patients 2-month of GH-withdrawal caused significant changes in body composition, including decreased fat-free mass, skeletal muscle mass, total body water, and intracellular water content (all, p<0.01); but these changes almost fully recovered 1 month after reinstituting GHRT. Unexpectedly, afamin levels decreased after GH-withdrawal (p=0.03) and increased with reinstitution (p<0.01). Changes of afamin levels during GH-withdrawal positively correlated with changes of HOMA-IR (r=0.80; p<0.01) and changes of insulin (r=0.71; p=0.02).

**Conclusion:**

Higher afamin levels in unsubstituted AGHD patients might indicate severe metabolic dysregulation. Significant changes accompanying GH-withdrawal and reinstitution, along with strong correlations with measures of IR, suggest that afamin could be a promising biomarker to monitor GHRT-associated changes of insulin sensitivity.

## Graphical Abstract

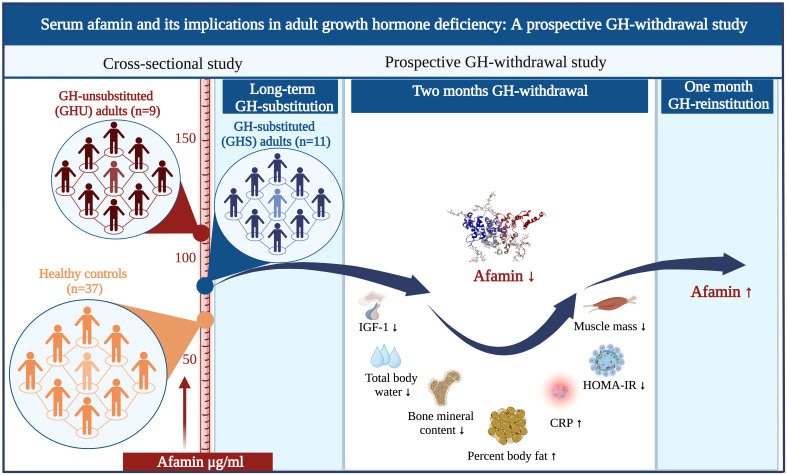

## 1 Introduction

Growth hormone (GH) deficiency in adults is characterized by an unfavorable cardiometabolic risk profile (1) and shares common features with metabolic syndrome (MS) (2). In fact, both treated and untreated adult GH-deficiency (AGHD) are associated with increased prevalence of MS (3), which has been suggested to contribute to the increased cardiovascular morbidity (4, 5). Supporting this, AGHD patients with MS were reported to have higher prevalence of coronary and cerebrovascular morbidity compared to patients with no MS (5).

Although the impact of MS on cardiometabolic risk appears obvious, the use of MS criteria to determine the severity of cardiometabolic risk in AGHD patients is highly debatable, because the cardiometabolic risk related to MS has been established in the general population, not in patients with hypopituitarism (3, 6, 7). Further, the insulin-like growth factor-1 (IGF-1) levels are not always related to the severity of metabolic dysregulation in AGHD (8). These controversies point to a growing need for biomarkers to evaluate individual cardiometabolic risk and to assess responses to growth hormone replacement therapy (GHRT) in AGHD patients (9).

Afamin is a liver-derived glycoprotein discovered in 1994 (10). Since then, it has been identified as a potential biomarker for pregnancy-related complications, neurological pathologies, and various types of cancer (11). Increased serum afamin level was found to be associated with insulin resistance (IR), MS, and type 2 diabetes mellitus (T2DM) (12–14). Moreover, afamin has been suggested to be a potential biomarker for the early identification of patients with high risk of T2DM (13).

In addition to predisposing to MS, both treated and untreated AGHD have been linked to disturbances of glucose metabolism (5, 15). However, afamin has not been investigated in these patients. Therefore, we aimed to study serum afamin and its correlation with anthropometric and routine laboratory parameters in GH-substituted (GHS) and GH-unsubstituted (GHU) patients as well as in healthy controls. Furthermore, we performed a prospective GH-withdrawal study in which afamin along with anthropometric, body composition and biochemical parameters were determined after a two-month GH-withdrawal and after GH-reinstitution.

## 2 Materials and methods

### 2.1 Participants

#### 2.1.1 Growth hormone deficient subjects

From May 2021 to May 2023, a total of 20 AGHD patients (11 GHS and 9 GHU patients) with an established diagnosis of AGHD were recruited form the outpatient clinic of our Endocrinology Unit. The diagnosis of AGHD was based on a peak serum GH response to insulin tolerance test less than 3 µg/L when adequate hypoglycemia (blood glucose lower than 2.2 mmol/L) was achieved (16). At the time of enrolment, each GHS patient received stable GH replacement for at least a year. GHU subjects were either GH naive or received no GH substitution for at least 2 years before study entry. In two GHU subjects GHRT was not initiated because of safety concerns (e.g., risk of tumor recurrence or progression), while seven patients stopped replacement therapy due to side effects (n=2) or lack of perceived positive effects (n=5). Exclusion criteria included active malignancy, heart failure, kidney failure, liver cirrhosis, pregnancy, breastfeeding and inability to comply with the study protocol. Each enrolled patient had multiple pituitary hormone deficiencies. At study entry all concomitant pituitary deficiencies were adequately substituted. Gender, age, the proportion of childhood-onset GH-deficient and T2DM patients did not differ significantly between the GHS and GHU groups. Clinical characteristics of AGHD patients are presented in **Table 1**.

**Table 1:** Clinical characteristics of patients with AGHD.

	GHS patients (n=11)	GHU patients (n=9)
Male/female	6/5	5/4
Age, years mean(range)	43.18 (26-59)	42.22 (21-61)
Patients with CO pituitary disease (n)	5	3
Duration of GHRT, years mean (range)	18.73 (4-43)	
Current GH dose mg/d mean(range)	0.31 (0.1-0.6)	
Etiology (n)		
Non-functioning pituitary adenoma	1	2
Functioning pituitary adenoma	1	1
Craniopharyngioma	1	4
Empty sella	3	1
Idiopathic	2	0
Other*	3	1
Cranial surgery (n)	4	8
Cranial irradiation (n)	2	1
Concomitant Hormonal deficiencies (n)		
TSH deficiency	11	9
ACTH deficiency	8	8
LH/FSH deficiency	7	8
ADH deficiency	5	3
Other relevant medical conditions (n)		
T2DM	2	2
T1DM	1	0
Hypertension	1	1
Osteopenia	3	4
Further medications (n)		
Bromocriptine	1	1
Beta-blocker	1	1
ARB	0	1
Statin	0	2
Fibrate	0	2
Ezetimibe	2	2
Metformin	2	1
GLP-1-RA	0	1
SGLT2i	0	1
Insulin	1	0
Oral anticoagulant	2	0

^*^Sheehan’s syndrome, surgery and radiotherapy due to ependymoma (n=1), and astrocytoma (n=2). ACTH, adrenocorticotropic hormone; ADH, antidiuretic hormone; AGHD, adult growth hormone deficiency; ARB, angiotensin receptor II blocker; CO, childhood-onset; FSH, follicle stimulating hormone; GHRT, growth hormone replacement therapy; GLP-1-RA, glucagon-like peptide 1 receptor agonist; GHS, growth hormone substituted GH-deficient patients; GHU, GH-unsubstituted GH-deficient patients; LH, luteinizing hormone; SGLT2i, sodium-glucose cotransporter-2 inhibitor; T1DM, type 1 diabetes mellitus; T2DM, type 2 diabetes mellitus; TSH, thyroid stimulating hormone.

#### 2.1.2 Healthy control subjects

Thirty-seven age- and gender-matched healthy volunteers, employees of the National Ambulance Service, served as controls.

### 2.2 Study design

#### 2.2.1 Cross-sectional study

In the cross-sectional study, both AGHD patients (n=20) and controls (n=37) underwent anthropometric measurements, body composition analysis, measurement of routine laboratory parameters as well as measurement of serum afamin concentrations. GHS patients (n=11) were invited to participate in the prospective GH-withdrawal study. After detailed explanation, all 11 GHS patients consented to participate in the study. For these 11 GHS patients, this visit was regarded as their baseline visit (Visit 1).

#### 2.2.2 Prospective GH-withdrawal study

GH-substitution of the 11 enrolled GHS patients was discontinued on the day of the baseline visit (Visit 1). All other hormone replacement therapies were continued unchanged during the study. After two months of GH-withdrawal, patients underwent the same examinations (Visit 2). Then, their pre-study GH doses were reinstituted, and the patients progressed to a 1-month GH-reinstitution period. One month later, while on GHRT (Visit 3), the same tests were repeated as in Visits 1 and 2, to assess the reversibility of changes induced by GH-withdrawal.

Patients were instructed to maintain their usual diet and physical activity throughout the study. The adequacy of hormone replacement therapy was assessed at each visit by measuring serum cortisol, thyroxine, testosterone, and electrolyte levels. Besides measuring IGF-1 levels at each visit, compliance was also evaluated by regular phone calls. Enrolled patients were not provided with prescriptions for their usual recombinant human GH (rhGH) preparations during the withdrawal period. All patients completed the 3 visits and remained on GHRT after finishing the study.

### 2.3 Ethics

Ethical approval was obtained from the Regional Ethics Committee of the University of Debrecen (registration number: RKIB/IKEB 5576-2020). All participants provided written informed consent and the study was conducted in accordance with the Declaration of Helsinki.

### 2.4 Measurements

#### 2.4.1 Anthropometric measurements

Measurements of height, weight, and waist circumference were obtained for anthropometric analysis. Body weight was measured during body composition analysis. Standing height was measured in centimeters with a calibrated Harpenden stadiometer. BMI was calculated as body weight (kg)/height (m^2^). Waist circumference (WC) was measured at the midline between the rib cage and the iliac crest, while hip circumference at the maximum circumference at the level of the femoral trochanters, both in the standing position. Waist-hip ratio (WHR) was calculated as WC (cm)/hip circumference (cm).

#### 2.4.2 Body composition analysis

Body composition was analyzed using multi-frequency bioelectrical impedance analysis (BIA) (InBody720, Inbody Co., LTD, Seoul, Korea). Testing was performed according to the manufacturer’s instructions. Participants were instructed to wear light clothes and arrive at the laboratory following an overnight fast. The following body composition parameters were measured: body fat mass, percent body fat, fat free mass, skeletal muscle mass, visceral fat area, total body water, extracellular water content, intracellular water content and bone mineral content.

#### 2.4.3 Measurement of routine laboratory parameters

Venous blood samples were collected into Vacutainer^®^ tubes (Becton Dickinson, San Jose, CA, USA) after an overnight fast. Sera and ethylenediaminetetraacetic acid (EDTA) anticoagulated plasma samples were centrifuged after 30 min at 3500 g, 15 mins, +4°C. Routine laboratory parameters, including high sensitivity C-reactive protein (hsCRP) fasting glucose, insulin, C-peptide, estimated glomerular filtration rate (eGFR), aspartate aminotransferase (AST), lipid parameters: triglyceride, total cholesterol, low-density lipoprotein-cholesterol (LDL-C), high-density lipoprotein-cholesterol (HDL-C), supersensitive thyroid stimulating hormone (sTSH), thyroxine, cortisol, testosterone and IGF-1 measurements were performed with a Cobas c600 autoanalyzer (Roche Ltd., Mannheim, Germany) with standard laboratory techniques from the same vendor at the Department of Laboratory Medicine, Faculty of Medicine, University of Debrecen. HOMA-IR was calculated by (fasting insulin x fasting glucose)/22.5 (17). Sera were kept at -70°C until afamin measurements.

#### 2.4.4 Measurement of afamin

Serum afamin levels were measured by a commercially available ELISA kit (Afamin Human ELISA, cat. number: RD194428100R, BioVendor, Brno, Czech Republic) according to the manufacturer’s instructions. The intra- and inter assay variation coefficients were <3.61% and <3.4%, respectively. Samples were used in 100-fold dilution.

### 2.5 Statistical analysis

Data were expressed as mean ± SD or median ± interquartile range unless otherwise specified. Statistical analyses were performed using Statistica 13.5.0.17 software (TIBCO Software Inc., Tulsa, OK, USA). Graphs were prepared using GraphPad Prism 9.4.1 (GraphPad Prism Software Inc., San Diego, CA, USA). The normality of data was checked using Kolmogorov–Smirnov and Shapiro-Wilk tests. Nonnormally distributed data were transformed logarithmically before analysis. Comparison of baseline parameters between groups was performed using one-way ANOVA with Tukey’s *post hoc* test. The effect of GH-withdrawal was analyzed using repeated measures ANOVA with Tukey’s *post hoc* test. To avoid problems of non-sphericity, Greenhouse-Geisser correction was used for all variables. When normal distribution could not be reached by logarithmic transformation, Kruskal-Wallis test and Friedman-test were performed. Differences between continuous variables were calculated using Chi-square test and Fisher’s exact test. Pearson’s and Spearman’s correlation were used to explore associations between selected variables. P ≤0.05 was considered statistically significant.

## 3 Results

### 3.1 Cross-sectional study

**Figure 1:**
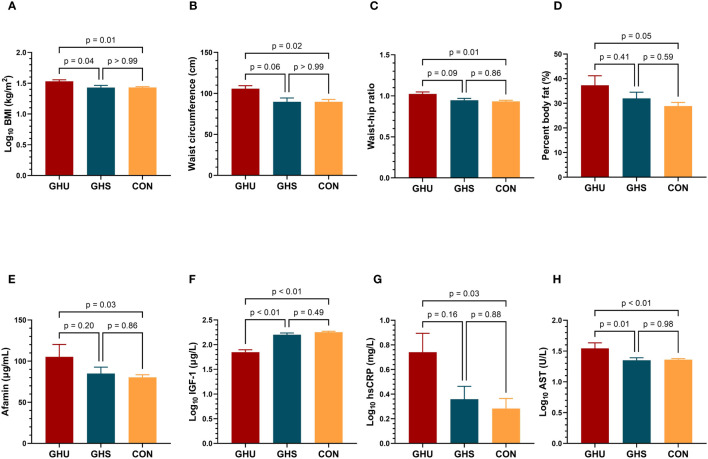
Comparison of **(A)** BMI; **(B)** waist circumference; **(C)** waist-hip ratio; **(D)** percent body fat **(E)** afamin concentrations; **(F)** IGF-l levels; **(G)** hsCRP; **(H)** AST; between GH-unsubstituted GH-deficient patients (GHU; dark red bars, n=9), GH-substituted GH-deficient patients (GHS; blue bars; n=11) and healthy control subjects (CON; yellow bars; n=37). Comparisons were carried out using one-way ANOVA. Bars represent means and error bars represent SEM. P values derive from Tukey’s *post hoc* test. AST, aspartate aminotransferase; BMI, body mass index; CON, healthy control subjects; GHS, GH-substituted GH-deficient subjects; GHU, GH-unsubstituted GH-deficient subjects; hsCRP, high sensitivity C-reactive protein; IGF-1, insulin-like growth factor 1.

Main anthropometric, body composition and biochemical parameters of the study groups are summarized in **Table 2**. GHU patients had higher BMI than GHS and control subjects (p=0.04 and p=0.01, respectively), whereas BMI was comparable between GHS and controls (**Figure 1A**). WC, WHR and percent body fat were also found significantly higher in GHU compared to control subjects (p=0.02; p=0.01 and p=0.05, respectively) (**Figures 1B–D**).

**Table 2:** Anthropometric, body composition and laboratory parameters of the patients and controls.

	GHU (n=9)	GHS (n=11)	CON (n=37)	p values
Male/female (n)	5/4	6/5	17/20	ns
Age (years)	42.2 ± 14.6	43.2 ± 10.2	47.6 ± 10.6	ns
Anthropometry and body composition				
Height (cm)	168.8 ± 14.6	163.7 ± 10.9	170.6 ± 10.3	ns
Weight (kg)	91.4 ± 25.8	76.2 ± 27.0	80.9 ± 19.4	ns
Body mass index (kg/m^2^)	32.4 (28.8-39.6)*^,#^	27.8 (21.6-36.0)	27.1 (22.9-29.3)	0.04* 0.01^#^
Waist circumference (cm)	105.8 ± 10.9^#^	89.8 ± 15.2	89.9 ± 16.1	0.02^#^
Waist-hip ratio	1.02 ± 0.08^#^	0.95 ± 0.08	0.93 ± 0.08	0.01^#^
Percent body fat (%)	37.3 ± 11.6^#^	32.0 ± 8.4	28.9 ± 8.9	0.05^#^
Visceral fat area (cm^2^)	153.5 ± 28.5	130.5 ± 51.6	127.8 ± 53.0	ns
Laboratory parameters				
Afamin (µg/mL)	105.2 ± 45.2^#^	85.0 ± 25.5	80.3 ± 19.2	0.03^#^
IGF-1 (µg/L)	67.5 (53.6-96.5)*^,#^	162.0 (146.0-180.0)	185.2 (153.2-223.4)	<0.01* <0.01^#^
hsCRP (mg/L)	3.4 (2.7-12.2)^#^	2.1 (1.4-3.5)	1.6 (0.7-3.0)	0.03^#^
Glucose (mmol/L)	4.8 (4.3-5.5)	5.0 (4.7-5.7) (n=10)	5.0 (4.7-5.3)	ns
C-peptide (pmol/L)	1253 (675-1710)	1096 (916-1410) (n=10)	1180 (798-1813)	ns
Insulin (mU/L)	23.6 (9.9-56.5)^#^	19.9 (9.7-54.1) (n=10)^§^	14.9 (8.1-34.1)	0.03^#^ 0.05^§^
HbA1C (%)	5.4 (5.2-5.9)	5.4 (5.2-6.5) (n=10)	5.4 (5.2-5.6)	ns
HOMA-IR	4.7 (2.1-12.3)	4.5 (2.2-12.6) (n=10)^§^	2.7 (1.5-4.8)	0.05^§^
eGFR (mL/1.73 m^2^)	90.0 (83.5-90.0)	90.0 (78.0-90.0)	83 (73.0-90.0)	ns
AST (U/L)	30.0 (22.0-68.5)*^,#^	25.0 (17.0-29.0)	22.5 (20.0-26.0)	0.01* <0.01^#^
Thyroxine (pmol/L)	15.7 ± 4.4	16.9 ± 4.9	15.4 ± 2.1	ns
Cortisol (nmol/L)	126.5 (49.9-267.7)	172.1 (57.4-332.8)	86.2 (63.0-115.1)	ns
Triglyceride (mmol/L)	2.3 (1.5-3.6)	1.7 (1.2-2.6)	1.7 (1.1-2.4)	ns
Total cholesterol (mmol/L)	5.1 ± 1.4	5.3 ± 0.7	5.8 ± 1.0	ns
HDL-C (mmol/L)	1.1 ± 0.3	1.2 ± 0.3	1.4 ± 0.4	ns
LDL-C (mmol/L)	3.2 ± 0.9	3.0 ± 0.6	3.4 ± 0.7	ns

Data are expressed as mean ± SD or median ± interquartile range in case of nonnormally distributed data and analyzed using one-way ANOVA. P values derive from Tukey’s *post hoc* test and are presented if the overall ANOVA has a p value of less than or equal to 0.05. Difference of male/female ratio between groups was analyzed using Chi-square test and Fisher’s exact test. Our T1DM patient in the GHS group has been excluded from the analysis of the parameters of glucose metabolism. ^*^Indicates statistically significant difference between GHU and GHS. ^#^Indicates statistically significant difference between GHU and CON. ^§^Indicates statistically significant difference between GHS and CON. AST, aspartate aminotransferase; CON, healthy control subjects; eGFR, glomerular filtration rate; GHS, GH-substituted GH-deficient patients; GHU, GH-unsubstituted GH-deficient patients; HbA1C, Hemoglobin A1C; HDL-C, high density lipoprotein cholesterol; HOMA-IR, homeostatic model assessment for insulin resistance; hsCRP, high sensitivity C-reactive protein; IGF-1, insulin-like growth factor 1; LDL-C, low density lipoprotein cholesterol; ns, not significant.

Mean afamin concentration was 31% higher in GHU compared to controls (p=0.03), while no significant difference was detected between GHU and GHS (**Table 2**, **Figure 1E**). Serum IGF-1 levels were lower in GHU compared to both GHS and control group. As a result of adequate GH-substitution, IGF-1 was similar in GHS and control subjects (**Table 2**, **Figure 1F**). hsCRP was also found higher in GHU than in control subjects (p=0.03) but the difference between GHU and GHS did not reach significance (**Table 2**, **Figure 1G**). Both GHU and GHS demonstrated higher insulin (p=0.03; p=0.05, respectively) and HOMA-IR (p=0.05; p=0.05, respectively) compared to controls. Serum AST concentrations were higher in GHU compared to GHS and controls (p=0.01, p<0.01, respectively) (**Table 2**, **Figure 1H**). Fasting glucose, C-peptide, HbA1C, eGFR, thyroxine, cortisol levels and lipid parameters (triglyceride, total cholesterol, HDL-C, LDL-C) were not different in the three groups (**Table 2**).

**Figure 2:**
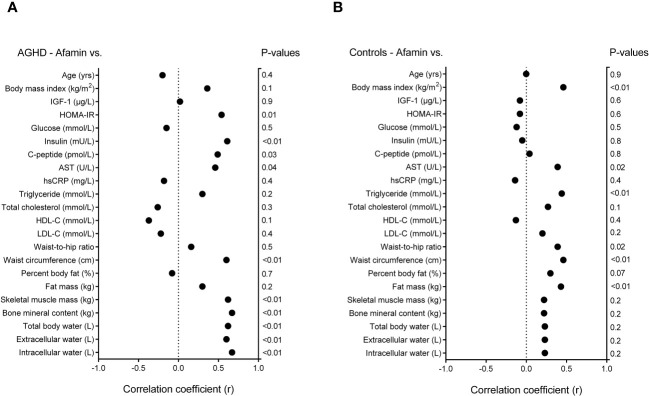
Data of Pearson’s correlations of serum afamin with main anthropometric, laboratory and body composition parameters **(A)** in patients with adult growth hormone deficiency (n=20); and **(B)** in age, gender and body mass index matched healthy controls (n=37). AGHD, adult growth hormone deficiency; hsCRP, high sensitivity C-reactive protein; HDL-C, high-density lipoprotein-cholesterol; HOMA-IR, homeostatic model assessment for insulin resistance; IGF-1, insulin-like growth factor-1; LDL-C, low-density lipoprotein-cholesterol.

When correlations between afamin and selected variables were calculated, all AGHD patients, including GHU and GHS patients were considered as a single AGHD cohort. Data from Pearson’s correlations of serum afamin with main anthropometric, laboratory and body composition parameters in patients with AGHD (n=20) and in healthy controls (n=37) are presented in **Figure 2**. In AGHD patients, but not in controls, afamin showed strong positive correlations with skeletal muscle mass, bone mineral content, total body water, extracellular- and intracellular water content (p<0.01). In AGHD, afamin was positively correlated with HOMA-IR (p=0.01), insulin (p<0.01) and C-peptide (p=0.03) levels, while in the control group afamin did not correlate with any of the parameters of glucose metabolism. Afamin correlated positively with triglyceride (p<0.01) levels, BMI (p<0.01), WHR (p=0.02) and fat mass (p<0.01) in controls, but not in AGHD patients. Both in AGHD and control subjects, afamin showed positive correlations with AST levels (p=0.04 and p=0.02, respectively) and WC (p<0.01 and p<0.01, respectively). Serum IGF-1 concentrations did not correlate with afamin levels either in AGHD subjects or in controls.

### 3.2 Prospective GH-withdrawal study

**Figure 3:**
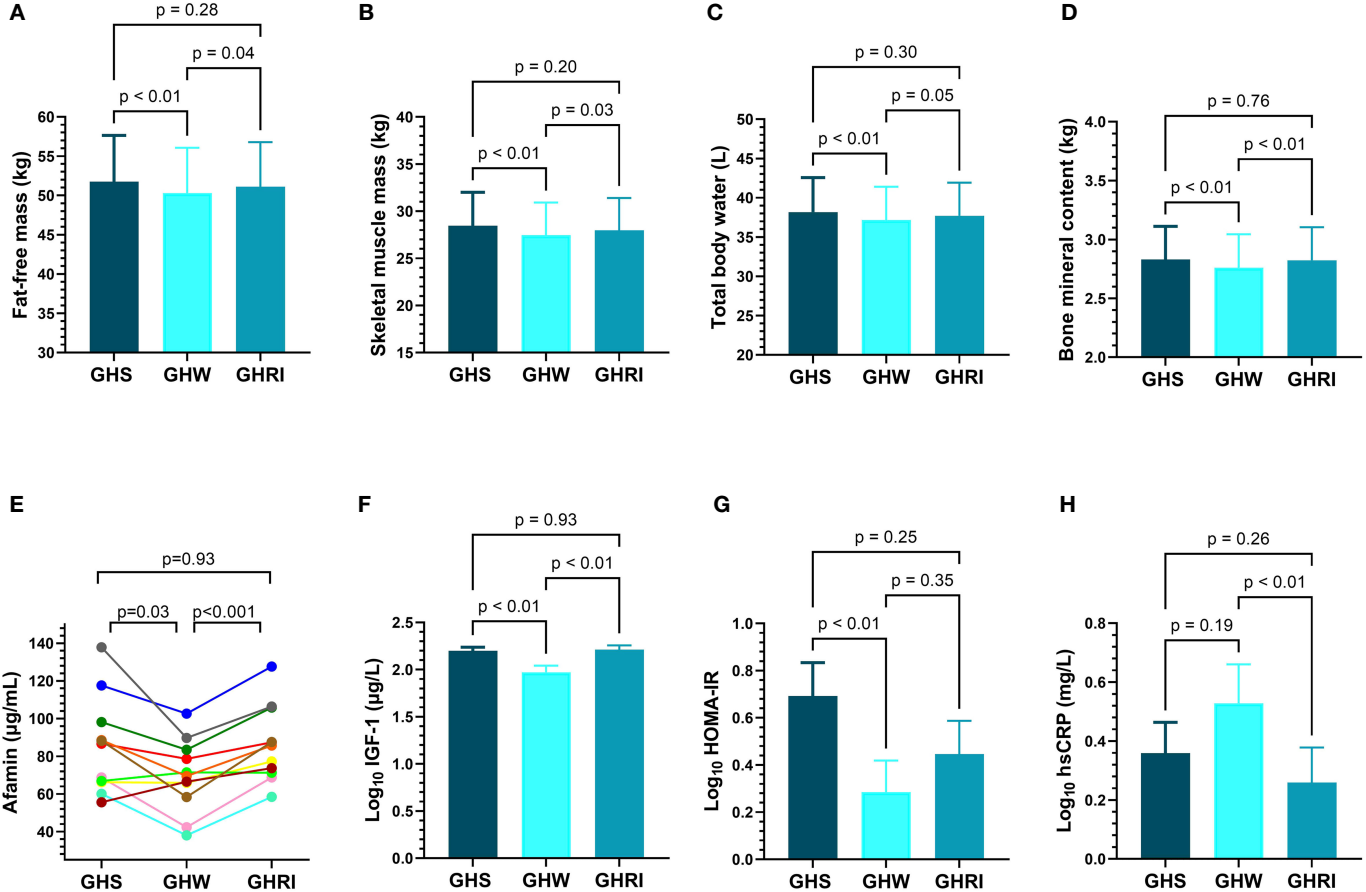
Changes of **(A)** fat-free mass; **(B)** skeletal muscle mass; **(C)** total body water; **(D)** bone mineral content **(E)** individual afamin levels; **(F)** IGF-1; **(G)** HOMA-IR; **(H)** hsCRP; in AGHD patients during long-term GH-substitution (GHS; dark blue; n=11), after two-month of GH-withdrawal (GHW; light blue; n=11) and following one month of GH-reinstitution (GHRI; medium blue; n=11). Comparisons were carried out using repeated measures ANOVA. Bars represent means and error bars represent SEM. P values derive from Tukey’s *post hoc* test. AGHD, adult growth hormone deficiency; GHRI, growth hormone reinstitution; GHS, GH-substituted GH-deficient subjects; GHW, growth hormone withdrawal; HOMA-IR, homeostatic model assessment for insulin resistance; hsCRP, high sensitivity C-reactive protein; IGF-1, insulin-like growth factor 1.  .

Anthropometric, body composition and laboratory parameters of AGHD patients on long-term GH-substitution (GHS), after 2-month of GH-withdrawal (GHW), and 1-month following GH-reinstitution (GHRI) are presented in **Table 3**. Two-month of GH-withdrawal did not result in significant changes in the anthropometric parameters, including body weight, BMI, WC and WHR in GHS subjects. Percent body fat showed a slight increase after 2-month of GH-withdrawal (mean difference: 1.73%, p=0.04) and did not return to baseline following 1-month reinstitution (**Table 3**). GH-withdrawal resulted in a substantial decrease in the fat-free mass, skeletal muscle mass, total body water and bone mineral content, but all of them returned nearly to baseline after 1-month of GH-reinstitution (**Table 3**, **Figures 3A–D**). Visceral fat area, fat mass and extracellular water content did not change significantly following GH-withdrawal (**Table 3**).

**Table 3:** Anthropometric, body composition and laboratory parameters of AGHD patients while on long-term GH-substitution (GHS), after 2-month GH-withdrawal (GHW), and 1 month after GH-reinstitution (GHRI).

	GHS (n=11)	GHW (n=11)	GHRI (n=11)	p values
	(=Visit 1)	(=Visit 2)	(=Visit 3)	
Anthropometry				
Weight (kg)	76.2 ± 27.0	75.7 ± 25.4	76.9 ± 25.7	ns
Body mass index (kg/m^2^)	25.6 (21.6-36.0)	25.3 (23.3-35.7)	25.7 (23.6-36.3)	ns
Waist circumference (cm)	89.8 ± 15.2	90.2 ± 12.8	89.6 ± 13.5	ns
Waist-hip ratio	0.95 ± 0.08	0.95 ± 0.07	0.95 ± 0.07	ns
Body composition				
Percent body fat (%)	32.0 ± 8.4*	33.7 ± 8.5	33.5 ± 8.6	0.04*
Visceral fat area (cm^2^)	130.5 ± 51.6	134.2 ± 43.1	131.60 ± 44.6	ns
Fat mass (kg)	24.5 ± 11.1	25.4 ± 10.4	25.8 ± 11.0	ns
Fat-free mass (kg)	51.8 ± 19.6*	50.3 ± 19.1^#^	51.1 ± 18.9	<0.01* 0.04^#^
Skeletal muscle mass (kg)	28.5 ± 11.8*	27.5 ± 11.5^#^	28.0 ± 11.3	<0.01* 0.03^#^
Total body water (L)	38.2 ± 14.5*	37.2 ± 14.1	37.7 ± 14.0	<0.01*
Intracellular water content (L)	23.4 ± 9.0*	22.6 ± 8.8^#^	23.0 ± 8.7	<0.01* 0.03^#^
Extracellular water content (L)	14.8 ± 5.5	14.6 ± 5.3	14.7 ± 5.3	ns
Bone mineral content (kg)	2.83 ± 0.9*	2.76 ± 0.9^#^	2.82 ± 0.9	<0.01* <0.01^#^
Laboratory parameters				
Afamin (µg/mL)	85.0 ± 25.5*	69.7 ± 19.1^#^	86.4 ± 20.1	0.03* <0.01^#^
IGF-1 (µg/L)	162.0 (146.0-180.0)*	95.1 (67.4-141.7)^#^	164.3 (134.3-222.7)	<0.01* <0.01^#^
hsCRP (mg/L)	2.1 (1.4-3.5)	3.6 (1.4-8.2)^#^	1.3 (1.2-4.0)	<0.01^#^
Glucose (mmol/L)	5.0 (4.7-5.7)	4.7 (4.0-5.2)	4.9 (4.4-5.1)	ns
C-peptide (pmol/L)	1096 (915-1410)* (n=10)	731 (424-1148) (n=10)	807 (532-1670) (n=10)	0.04*
Insulin (mU/L)	19.9 (9.7-54.1)* (n=10)	11.1 (7.5-14.9) (n=10)	16.6 (6.1-22.3) (n=10)	0.01*
HbA1C (%)	5.4 (5.2-6.5) (n=10)	5.5 (5.1-6.2) (n=10)	5.5 (5.1-6.3) (n=10)	ns
HOMA-IR	4.5 (2.2-12.6)* (n=10)	2.3 (1.4-3.4) (n=10)	3.8 (1.2-5.7) (n=10)	<0.01*
eGFR (mL/1.73 m^2^)	90.0 (78.0-90.0)	90.0 (82.0-90.0)	90.0 (90.0-90.0)	ns
AST (U/L)	25.0 (17.0-29.0)	20.0 (17.0-35.0)	21.0 (17.0-29.0)	ns
Thyroxine (pmol/L)	16.9 ± 4.9	18.6 ± 2.7	17.3 ± 2.6	ns
Cortisol (nmol/L)	172.1 (57.4-332.8)	232.9 (157.0-413.1)	136.0 (106.1-271.7)	ns
Triglyceride (mmol/L)	1.7 (1.2- 2.6)	1.6 (1.1-2.2)	1.6 (1.2-1.8)	ns
Total cholesterol (mmol/L)	5.3 ± 0.7	4.9 ± 0.9	5.3 ± 0.8	ns
HDL-C (mmol/L)	1.2 ± 0.3	1.5 ± 0.3	1.4 ± 0.2	ns
LDL-C (mmol/L)	3.0 ± 0.6	3.1 ± 0.9	3.1 ± 0.6	ns
Hemoglobin (g/L)	155.6 ± 12.4	149.7 ± 12.0	149.1 ± 10.0	ns
Hematocrit	0.46 ± 0.04	0.45 ± 0.03	0.45 **±** 0.03	ns

Data are expressed as mean ± SD or median and interquartile range in case of nonnormally distributed data and analyzed using repeated measures ANOVA. Greenhouse-Geisser correction was applied to correct any potential problems of non-sphericity. P values derive from Tukey’s *post hoc* test and are presented if the overall ANOVA has a p value of lower than or equal to 0.05. Our T1DM patient has been excluded from the analysis of the parameters of glucose metabolism. ^*^ Indicates statistically significant difference between GHS and GHW. ^#^ Indicates statistically significant difference between GHW and GHRI. No statistically significant difference was found between GHS and GHRI. AGHD, adult growth hormone deficiency; AST, aspartate aminotransferase; eGFR, glomerular filtration rate; GHRI, growth hormone reinstitution; GHS, growth hormone substitution; GHW, growth hormone withdrawal; HbA1C, Hemoglobin A1C; HDL-C, high density lipoprotein cholesterol; HOMA-IR, homeostatic model assessment for insulin resistance; hsCRP, high sensitivity C-reactive protein; IGF-1, insulin-like growth factor 1; LDL-C, low density lipoprotein cholesterol; ns, not significant.

As expected, serum IGF-1 concentrations declined following GH-withdrawal (p<0.01) and then increased (p<0.01) after reinstituting GHRT (**Figure 3F**). Interestingly, afamin levels also showed a significant decrease after 2-month of GH-withdrawal (p=0.03) and then returned to baseline following GH-reinstitution (**Figure 3E**). C-peptide, insulin and HOMA-IR also decreased following GH-withdrawal, but they did not return to baseline after 1-month of retreatment (**Table 3**, **Figure 3G**). The rise in hsCRP after GH-withdrawal did not reach statistical significance, but reinstitution of GHRT resulted in a decrease (p<0.01) in the hsCRP levels (**Table 3**, **Figure 3H**). AST, eGFR, thyroxine, cortisol, hemoglobin, hematocrit levels and parameters of lipid-metabolism remained unchanged throughout the study (**Table 3**). The change of afamin (Δafamin) = (afamin after GH reinstitution – afamin after withdrawal) was positively correlated with the change of HOMA-IR (ΔHOMA-IR; r=0.80; p<0.01) and the change of insulin (Δinsulin; r=0.71; p=0.02).

## 4 Discussion

In our cross-sectional study, afamin levels were 31% higher in GHU subjects compared to controls. Although one study has identified afamin as a potential marker to detect GH administration in healthy athletes (18), to the best of our knowledge, this is the first study to investigate afamin in AGHD patients. Even though the physiological properties of this hepatokine are not fully characterized, increasing evidence has shown that afamin is strongly associated with MS (12), T2DM (13), non-alcoholic fatty liver disease (NAFLD) (19) and other IR-related conditions (11, 14). In a large-scale study, each 10 mg/dL increment in afamin levels resulted in a 19% increase in the number of MS components (12). Importantly, mean afamin concentration in GHU subjects was found comparable to that of reported in morbidly obese T2DM subjects (105.2 vs. 109.2 μg/mL) (20), which might reflect the severity of metabolic dysregulation in GHU patients. Higher BMI, WC, WHR and fat mass found in GHU subjects could theoretically explain the higher afamin concentrations, however, afamin correlated only with WC among these parameters in AGHD. Interestingly, AST, a common marker of liver injury (21), was also found higher in GHU subjects, and consistent with previous studies, positively correlated with afamin levels (19, 22, 23). A former study with larger sample size also detected elevated AST levels (39.3 ± 28.4 IU/L) in unsubstituted AGHD patients. Explaining this finding, the prevalence of NAFLD was proved to be much higher in the unsubstituted AGHD group compared to controls (77 vs 12%, p<0.001) (24). Based on previous studies, afamin concentration is associated with hepatic fat content (23) and independently predict the development of NAFLD (19). Consequently, on the basis of literature data (24–26), it is reasonable to assume that higher AST along with higher afamin levels indicate higher rates of NAFLD in our GHU subjects. However, it remains only a speculation at this time because the presence of NAFLD was not tested in our study.

In AGHD, afamin showed positive correlations with several body composition parameters, including skeletal muscle mass, bone mineral content, intracellular as well as total body water content. Surprisingly, none of these parameters correlated with afamin in healthy controls. To date, only two studies have evaluated the association of afamin with body composition parameters: one of them detected no associations in normal-weight and obese pregnant women (27), while the other found positive correlation between afamin and lean mass in overweight and obese adults (23).

Osteopenia and increased risk of fracture are characteristic features of AGHD; therefore, bone mineral density and bone mineral content are monitored regularly in these patients (28, 29). Due to lack of knowledge and possible limitations, BIA is not used routinely to estimate bone mineral content in the clinical practice. However, in a recent study, total body bone mineral content measured by BIA and dual-energy X-ray absorptiometry (DXA) was found to be strongly correlated (r=0.83) (30). In our study, bone mineral content positively correlated with afamin levels (r=0.67, p<0.01) in AGHD patients. Unfortunately, no human study has investigated the role of afamin in bone metabolism, however, in a mouse model, afamin was demonstrated to induce a high bone turnover state and therefore suggested to be a potential biomarker for accelerated bone loss and osteoporotic fractures (31, 32).

Consistent with previous studies conducted on patients with various IR-related conditions (12, 13, 23), we detected positive correlations of afamin with HOMA-IR, insulin, and C-peptide levels in AGHD subjects. Even though the underlying mechanism is unclear, strong association of afamin with measures of IR and the finding of a hyperglycemic phenotype in mice transgenic the human afamin gene indicate a causative role of afamin in the development of T2DM (12, 19). This is also supported by the results of Shen et al. demonstrating a direct role of afamin in the glucose metabolism *in vitro* (33). Based on their findings, afamin can regulate the expression of several key enzymes of glucose metabolism (33). Untreated AGHD is associated with an increased prevalence of T2DM, largely because of the adverse body composition (34). According to an analysis including more than 20.000 individuals, afamin levels can predict the development of T2DM (13). Consequently, higher afamin levels together with higher insulin and HOMA-IR might represent high susceptibility to T2DM in GHU subjects. Several studies reported significant correlations between afamin and parameters of lipid metabolism (12–14, 35). In our study, afamin positively correlated with triglyceride levels in healthy controls, but significant associations were not found in AGHD.

In our prospective study, 2-month of GH-withdrawal did not result in changes of standard anthropometric parameters; but we found significant increase in percent body fat, and a significant decrease in fat-free mass, skeletal muscle mass, total body water, intracellular water content, and bone mineral content. Substantial changes of body composition without changes of BMI agrees well with previous results indicating that BMI is a poor indicator in monitoring GHRT, because the shift from fat to lean mass is not necessarily reflected in the BMI (36). Increased fat mass, a consistent finding of GH-withdrawal studies, was not detected in our study, but studies reporting increased fat mass used longer withdrawal period (3-18 months) (37). Our finding was consistent with the study of Kohno et al. who reported an increase in percent body fat two months after termination of GHRT, but the lean mass did not change significantly in their study (38). It should be mentioned that unlike most withdrawal studies, our study provides evidence that changes of body composition induced by short GH-withdrawal almost fully recover one month after reinstituting GHRT. In line with previous data (39, 40), we detected improved insulin sensitivity after GH-withdrawal. On the contrary, we did not find significant changes in the lipid parameters (39–41). As awaited, IGF-1 levels declined after withdrawal and then returned to baseline after GH reinstitution. Since even a short GH-withdrawal has been shown to adversely affect cardiometabolic risk factors (40), we expected higher afamin levels after GH-withdrawal. Surprisingly, afamin levels decreased significantly after GH-withdrawal and increased even more significantly during GH-reinstitution. Earlier studies revealed that hematocrit values have considerable effect on the plasma concentration of metabolites mainly distributed in the plasma (42). Since GH may influence erythropoiesis, it can be assumed that changes of afamin might be a consequence of the changes of hematocrit and hemoglobin values. However, in our prospective study, GH-withdrawal and reinstitution did not significantly modify hematocrit and hemoglobin values. As Δafamin showed strong positive correlations with ΔHOMA-IR and Δinsulin, it was revealed that the changes of afamin levels are largely attributable to the change of insulin sensitivity induced by GH-withdrawal and reinstitution. Although interventional studies investigating afamin levels are scarce, three studies have also linked the reduction of afamin levels to improved IR. Two of these studies detected decreased afamin levels after bariatric surgery (43, 44), while another study reported reduced afamin levels and improved insulin sensitivity in patients with MS after 2-month of treatment with an antidiabetic herbal product (45). Conversely, glucocorticoid therapy with its well-known negative effect on insulin sensitivity, is found to increase afamin levels (46).

As an interesting finding, we demonstrated that long-term GH-deficiency and short-term GH-withdrawal result in opposite effects on afamin concentrations. Although further research is required to clarify this phenomenon, our results clearly demonstrate that in short-term GH-withdrawal afamin levels are predominantly influenced by the cessation of the diabetogenic actions of rhGH therapy. On the other hand, considering the strong association of afamin with WC, AST levels and measures of IR, higher afamin levels in long-term GH-deficiency are presumably associated with abdominal obesity, consequent insulin resistance and NAFLD. As afamin levels in GHS patients were found comparable to that of healthy controls (85.0 ± 25.5 vs. 80.3 ± 19.2, p=0.86), our study also supports previous findings (1, 36) suggesting that GHRT could improve the cardiometabolic risk profile in patients with AGHD.

Measurement of afamin might have important clinical implications in the management of AGHD. Firstly, afamin could be utilized to monitor individual cardiometabolic risk in patients with either treated or untreated AGHD. Secondly, given the possible deteriorating effect of GHRT on IR (47), monitoring the glucose homeostasis during GHRT is essential, especially in patients with impaired glucose metabolism (29). Based on our findings, afamin appears to be a potential biomarker to monitor GHRT-associated changes of glucose homeostasis.

Notably, afamin has been demonstrated to exhibit several characteristics of an ideal biomarker (48). Dieplinger et al. demonstrated low variation of afamin over time, indicating a good suitability of afamin for serial measurement (48). Compared to certain currently used biomarkers, such as NT-proBNP and procalcitonin, reference change value of afamin was reported to be rather low (49, 50). Furthermore, unlike many of the currently used biomarkers of glucose homeostasis, afamin levels are independent of the prandial status (48). IGF-1 is currently used to monitor effects of GHRT; however, it is influenced by several biological factors, including age and sex and its relationship with clinically significant efficacy endpoints such as body composition, is very limited (51, 52). In contrast, afamin concentrations are not influenced by sex, age, and ethnicity (11), and according to our findings, it has strong associations with several body composition parameters, which are expected to change in response to GHRT.

Given that there is no predictive biomarker for cardiometabolic complications in AGHD, finding suitable markers to identify high risk patients is in the center of several studies. In a cross-sectional study, lipid accumulation product, which, just like afamin is a powerful marker of MS in the general population was also reported elevated in AGHD, and proved to have a strong diagnostic accuracy for MS in GH-deficient adults (53). In a long-term observational study Höybye et al. investigated the level of the inflammatory biomarker soluble urokinase plasminogen activator receptor (suPAR) in AGHD patients receiving GHRT. In former studies, elevated levels of suPAR were associated with progression and increased mortality in multiple patient populations. During long-term GHRT, levels of suPAR remained stable, which could be interpreted as a beneficial effect of hormone substitution. During the follow-up individual increases were also detected on occurrence of tumors or cardiovascular events, although, the number of patients was too small to draw conclusions (54). Besides identifying potential biomarkers, other studies pointed out that despite both MS and AGHD are associated with oxidative stress and chronic inflammation, the underlying pathology involved can be different in these disease processes. Accordingly, plasmatic lipocalin-2, a glycoprotein involved in several obesity-related conditions as well as chronic inflammatory processes was found to be elevated in MS but not in partial or total GHD (55). Moreover, the pattern of antioxidant defenses was also proved to be different in MS and AGHD (56, 57).

The main strength of our study is the prospective self-control design, which, despite the small number of participants, enabled us to detect significant changes after GH-withdrawal and reinstitution. Moreover, use of a one-month GH-reinstitution made it possible to provide evidence that the unfavorable changes of body composition induced by GH-withdrawal recover rapidly after reinstituting GHRT. Considering that even a metabolomic analysis investigating more than 200 metabolites failed to find potential markers to monitor GHRT (9), it should be emphasized that our study identified a promising biomarker, which is significantly modified by GH-withdrawal and reinstitution.

One limitation of our study is the small sample size; therefore, our results are considered preliminary and require confirmation in future studies. Further study with non-obese AGHD patients and larger number of unsubstituted AGHD patients also need to be performed to confirm that afamin is a disease-specific biomarker. However, it should be noted that AGHD is a rare endocrine disease and prospective studies especially GH-withdrawal studies, which can be associated with a risk of deterioration, often face recruitment difficulties. As a result, similarly small sample sizes are common among GH-withdrawal studies. Furthermore, in our cross-sectional study, when correlations of afamin were calculated, GHS and GHU patients were considered as a single AGHD cohort due to the small sample sizes. Thus, these results should be interpreted with caution. Additionally, in the healthy control group and the GHU group all measurements were performed only once, which is also a methodological limitation. Measurement of the serum afamin concentrations at the same time points for all groups would have allowed us to test the stability of afamin levels in a given individual. The use of BIA can also be considered a limitation, because it can be influenced by rapid changes of extracellular fluid volume, which can occur when GHRT is discontinued and reinstituted (58). On the other hand, the suitability of Inbody720 for body composition analysis has been confirmed by several validation studies in a wide range of populations (30).

In conclusion, our results support previous findings demonstrating that short-term GH-withdrawal induces alterations of body composition as well as improvement in IR in patients with AGHD. In addition, the present study demonstrated that these changes almost fully recover after one-month of treatment reinstitution. We observed higher afamin levels in untreated AGHD, which may confirm the presence of a severe metabolic dysregulation. Furthermore, our data provide preliminary evidence that afamin levels might be differently regulated in GH-deficient patients compared to healthy controls and show strong associations with several body composition parameters in AGHD. In patients receiving long-term GHRT, afamin concentrations were significantly modified by short-term GH-withdrawal and reinstitution suggesting that afamin could be a promising surrogate marker to monitor responses to GHRT.

## Data availability statement

The original contributions presented in the study are included in the article/supplementary material. Further inquiries can be directed to the corresponding author.

## Ethics statement

The studies involving humans were approved by Regional Ethics Committee of the University of Debrecen. The studies were conducted in accordance with the local legislation and institutional requirements. The participants provided their written informed consent to participate in this study.

## Author contributions

BR: Writing – original draft, Data curation, Formal analysis, Investigation, Visualization, Software. HL: Investigation, Software, Supervision, Writing – review & editing. SC: Data curation, Investigation, Writing – original draft. VS: Data curation, Investigation, Software, Writing – original draft. EB: Data curation, Investigation, Writing – original draft. MB: Conceptualization, Supervision, Writing – review & editing. EN: Conceptualization, Supervision, Writing – review & editing. ZS: Supervision, Writing – review & editing. MH: Supervision, Visualization, Writing – review & editing. SS: Conceptualization, Methodology, Supervision, Writing – review & editing.
